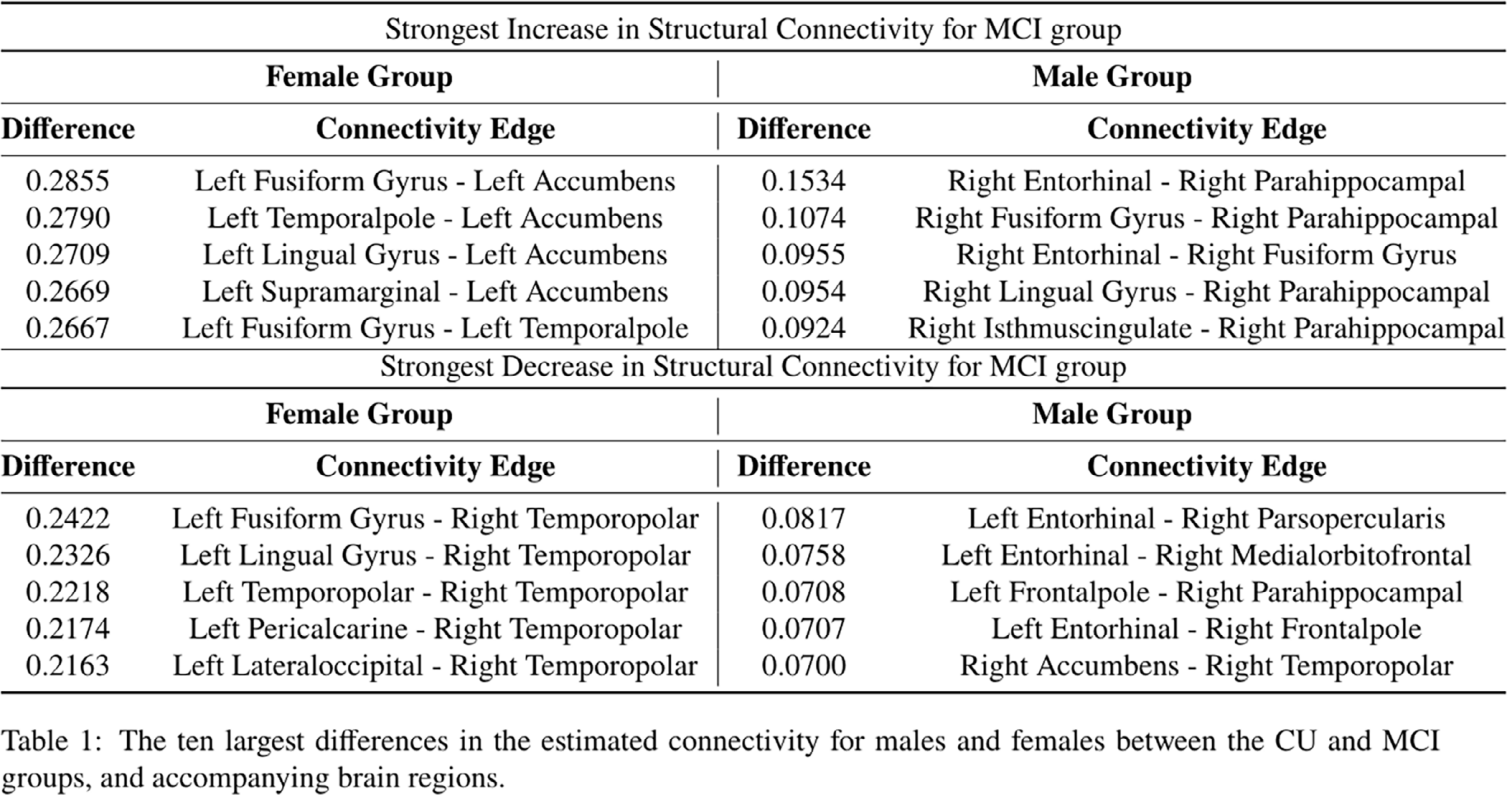# Sex differences in structural connectivity‐based AD pathology in the Indiana Memory and Aging cohort

**DOI:** 10.1002/alz70856_103077

**Published:** 2025-12-26

**Authors:** Connor Lee Cornelison, Qiuting Wen, Plamena P. Powla, Theyaneshwaran Jayaprakash, Shannon Risacher, Andrew J. Saykin, Yu‐Chien Wu, Selena Wang

**Affiliations:** ^1^ Indiana University, Bloomington, IN, USA; ^2^ Indiana University School of Medicine, Indianapolis, IN, USA; ^3^ Indiana Alzheimer's Disease Research Center, Indiana University School of Medicine, Indianapolis, IN, USA; ^4^ Indiana Alzheimer's Disease Research Center, Indiana University School of Medicine, Indianapolis, IN, USA; ^5^ Indiana University School of Medicine, Department of Radiology and Imaging Sciences, Indianapolis, IN, USA

## Abstract

**Background:**

Alzheimer's disease (AD) disproportionately impacts women. Roughly two‐thirds of U.S. adults experiencing AD related symptoms are women (Dement, 2023). Brain structural connectivity capturing the white matter fiber tract‐based connections among regions of the brain has been shown to provide a unique characterization of brain structural changes associated with AD (e.g., Shao et al., 2012). Orientation dispersion (OD) structural connectivity is a type of structural connection that reflects the neurite orientation fanning and variability of the brain fiber directions (Wen et al., 2019). In a prior study, Fu et al. (2020) established a potential link between loss of orientation dispersion connections and development of AD. We investigate differences in OD‐based structural connectivity between healthy participants and participants with Mild Cognitive Impairment (MCI).

**Method:**

Participant data (*n* = 100) were obtained from the Indiana Memory and Aging Study (IMAS) of the Indiana Alzheimer's Disease Research Center (IADRC) and included 71 females and 29 males, 78 cognitively unpaired (CU) and 22 MCI participants. We used a novel latent space framework (Wang et al., 2025) to detect differences between groups, by first estimating group‐level structural connectivity, and then identified differences between CU and MCI participants based on Bayesian inference.

**Result:**

In Table 1, we report the top ten connectivity edges found to be associated with MCI for males and females separately. Females showed significant changes in OD‐based structural connectivity with the development of MCI; the female group demonstrated significant differences (.2855, t=30.24, *p*¡0.005) between CU and MCI participants whereas the male groups did not show statistical significance in OD‐based structural connectivity (t=‐0.1631, *p* = .8705). Females with MCI showed consistent increases in the left accumbens connectivity compared to CU females. The right temporopolar region also showed consistent decrease in connectivity in the female MCI group compared to CU (0.2422, t=30.24, *p*¡0.005).

**Conclusion:**

Current literature supports the key role that both the accumbens (Guo et al., 2022) and temporopolar regions (Dautricourt et al., 2021) play in AD. We found differences between males and females in their structural connectivity‐based AD pathology, with women showing the left accumbens and right temporopolar regions consistently impacted by the development of MCI